# Drought and grazing drive the retrogressive succession by changing the plant–plant interaction of the main species in Inner Mongolia Steppe

**DOI:** 10.1002/ece3.4652

**Published:** 2018-11-20

**Authors:** Shaobo Gao, Zhirong Zheng, Yukun Wang, Lei Liu, Nianxi Zhao, Yubao Gao

**Affiliations:** ^1^ Department of Plant Biology and Ecology, College of Life Science Nankai University Tianjin China

**Keywords:** dominant species, drought stress, mowing disturbance, plant–plant interaction, typical steppe

## Abstract

Plant–plant interactions play a key role in the function and structure of communities. The combined effect of drought stress and grazing disturbance on shaping plant–plant interactions is still poorly understood, while this combination is common in semiarid ecosystems. Four species including *Stipa grandis*, which is dominant in the typical steppe, and *Stipa krylovii*,* Artemisia frigida,* and *Cleistogenes squarrosa*, which are dominant species in the *S. grandis* degraded communities, were selected as study targets. We conducted a competition experiment (uniformly dense monoculture or mixture, respectively) under controlled conditions, including both drought stress and mowing disturbance, and calculated the relative interaction index (RII) of tiller number and RII of biomass for each species under each condition. (a) Under the same condition, the RII of tiller number and that of biomass for the same species usually showed reverse trends. (b) Mowing disturbance rather than drought stress played a negative role in influencing *S. grandis*’ or *S. krylovii*’s RII of tiller number and played a positive role in influencing *A. frigida*'s RII of biomass. (c) Drought stress rather than mowing disturbance played a positive role in influencing *C. squarrosa*’s RII of tiller number. (d) Neighbor species significantly influenced *S. grandis*’ RII of tiller number, *S. krylovii*’s RII of tiller number, *A. frigida*'s RII of tiller number and biomass, and *C. squarrosa*’s RII of biomass. These results could provide an explanation for why *S. krylovii*,* A. frigida,* and *C. squarrosa* can replace *S. grandis* and become the dominant species when *S. grandis* communities undergo a process of degradation due to overgrazing or climatic drought in natural communities. The present study provided powerful evidences for species replacement in the typical steppe of Inner Mongolia and elucidated the driving mechanisms of *S. grandis* communities’ retrogressive succession.

## 1 INTRODUCTION

Understanding the process of retrogressive succession of plant community is very important in the restoration and protection ecology as well as in applied ecology (He & Bertness, 2014). Plant–plant interaction is thought to be the fundamental for determining community dynamic and one of the most fundamental issues in community ecology, especially how the balance between positive interaction (competition) and negative interaction (facilitation) responds to variation in environmental stress and disturbance (Bertness & Callaway, 1994; Brooker et al., 2008; Bruno, Stachowicz, & Bertness, 2003; Grime, 1974). The negative change of plant–plant interactions of dominant species could lead to being replaced by the prosperity of others (Soliveres & Maestre, 2014). Therefore, understanding how plant–plant interactions change with the environmental condition is the core to explain the community succession and to predict the community dynamics in the face of environmental changes.

The shift of competitive superiority from one species to another species would happen in stress or disturbance condition, which could cause the retrogressive successive. More recently, researchers have focused on understanding how species characteristics (ecotype, tolerance) and stress types affect competitive capacity in themselves. Although the tolerance or resistance of the species in the same community are thought to be similar, it shows difference when these species are under the stress or disturbance condition. On the other hand, many documents have reported that neighbor species could play an important role in affecting the competitive hierarchy of the target species (Ulrichet et al., 2016). That is to say, the plant–plant interaction could be positive if the target species grows with one species or negative if it grows with another species. As a result, the target species could keep its superiority with one species but lose its superiority with another species (Olsen, Topper, Skarpaas, Vandvik, & Klanderud, 2016). Even though the relationships between the characteristics (or stress types) of the target species and the result of the plant–plant interaction are complicated and may be species specific, it goes to the core of retrogressive succession. Therefore, fully understanding such relationships of the main species in a particular community is very important for ecologist.

The semiarid Inner Mongolian Steppe of China is an important part of the terrestrial ecosystem of the Eurasian steppe, and plant communities are often dominated by the tussock species *Stipa grandis* (Chen, Zhao, Zhang, & Gao, 2013; Lu & Wu, 1996). However, due to drought caused by climate change and overgrazing by cattle or/and sheep, a large area of grassland has become degraded. During the process of retrogressive succession, the dominant species *S. grandis* is gradually replaced by *Stipa krylovii* in the light degraded communities, by *Artemisia frigida* and *Cleistogenes squarrosa* in the heavy‐degraded communities. Accompanying with the replacement of dominant species is the loss of diversity and the steep decline in aboveground net primary productivity (Pan et al., 2016; Wan et al., 2015). Previous studies have focused more on water utilization and the life history to explore the species replacement during the degradation of *S. grandis* communities (Zhang, Zhu, Liu, & Pan, 2014). Researchers have tried to examine how drought or disturbance influenced the plant–plant interactions of the main species to provide some explanations for the species replacement in some other communities (Michalet et al., 2014; Smit, Rietkerk, & Wassen, 2009). Moreover, although some hypotheses, such as stress gradient hypothesis (SGH) or niche complementarity, could help us to predict how stress or disturbance affects the results of plant–plant interactions, they could not help us to efficiently predict how a combination of stress and disturbance shapes plant interactions (Barner, Hacker, Menge, & Nielsen, 2016; He, Bertness, & Altieri, 2013). Therefore, understanding how the combination of grazing disturbance and drought stress influences plant–plant interactions of the main species in Inner Mongolia Steppe of China is of critical importance for explaining the community retrogressive succession in this region, and guiding sustainable management practices of semiarid grassland ecosystems of China.

Therefore, in the present study, we selected the four species, *S. grandis, S. krylovii*,* A. frigida,* and *C. squarrosa*, as study subjects, and conducted a two‐factor (drought and mowing) isodensity displacement experiment to assess the variation of the relative interaction index (RII) between pairwise species under different conditions. Specifically, we intended to address the following questions. (a) How do drought stress and mowing disturbance (simulated grazing) affect the RII of each species? (b) Which characteristic of the species determine its RII under different conditions? (c) Are the RIIs of the same target species significantly influenced by its neighbor species? The results would propose an interpretation for the retrogressive succession of *S. grandis* communities in Inner Mongolia Steppe, China.

## 2 MATERIALS AND METHODS

### 2.1 Plant materials

Based on the retrogressive succession of *S. grandis* communities, we selected *S. grandis*,* S. krylovii*,* A. frigida,* and *C. squarrosa* as study subjects. Both *S. grandis* and *S. krylovii* (Poaceae) are perennial, dense bunch grasses with a large lemma. *A. frigida* (Asteraceae) is a perennial subshrub, which possesses powerful adventive roots, and *C. squarrosa* (Poaceae) is a C_4_ plant and widely distributes in Eurasian steppe. The seeds of *S. grandis, S. krylovii, A. frigida,* and *C. squarrosa* were collected in late August 2013 from Xilingol League, Inner Mongolia Autonomous Region, China, and preserved at 0°C.

### 2.2 Experimental design

We conducted a two‐factor (drought stress and mowing disturbance) isodensity displacement experiments, and each factor had two levels (present (+) and absent (−)). Soil moisture in the drought treatments (D+) was maintained at 8% ± 3%, which was equivalent to that of a semiarid grassland, while that in the nondrought treatments (D−) was kept at 15% ± 3% (Zhang et al., 2014). A soil compaction moisture tester (SCMT) was used to monitor the soil moisture every three days. Mowing was carried out to simulate moderate‐to‐heavy grazing disturbance, and the aboveground plant biomass was removed to a stubble height of 10 cm twice during the experiment.

We selected seedlings of equal size of the four species from seedling trays two weeks after germination and transplanted them into pots (10 cm in diameter and 20 cm in height) containing vermiculite (150 g) on 1st March 2014. According to the principle of replacement, there were ten types of planting, including 6 two‐species mixtures (*S. grandis*–*S. krylovii*,* S. grandis*–*A. frigida*,* S. grandis*–*C. squarrosa*,* S. krylovii*–*A. frigida*,* S. krylovii*–*C. squarrosa,* and *A. frigida*–*C. squarrosa*) and four monocultures. There were seven replicates per treatment, and we got 280 pots (10 × 2×2 × 7) in total. In mixtures, the one is the target species and the other is its neighbor species, therefore, each species had three different neighbor species per treatment. Mowing was performed on 1st May (2 months after plantation) and 1st July (4 months after plantation) 2014.

The study site was established in the experimental field at Nankai University in Tianjin (39°10N, 117°10E), China, where the mean annual air temperature is 12.6℃ and the mean annual precipitation over the last 40 years is 523.2 mm. The pots were placed under a rain‐proof shed to avoid natural precipitation. The shed was at a height of 5 m and could penetrate 95% natural light. The locations of the pots were randomly changed every month.

On 1st October 2014, we measured tiller number and harvested the plants by individual. The harvested individuals (including shoot and root) were dried at 70°C to a constant weight.

### 2.3 Data analysis

The relative interaction index (RII) of each target plant species was estimated by using the following formulas (Armas, Ordiales, & Pugnaire, 2004): $\text{RII}=\frac{P_{+N}-P_{-N}}{P_{-N}+P_{+N}}.$ *P_+__N_* represents the target species’ performance of tiller number (biomass) in mixture with a neighbor species under one treatment; *P*_‐N_ represents the target species’ average performance of tiller number (biomass) of an individual in monocultures under the same treatment. The RII of tiller number (biomass) was calculated according to three different neighbor species within each drought treatment (2) and disturbance treatment (2) cross‐treatment, totaling 12 RIIs of tiller number (biomass) for each target species. The RII varies from −1 to 1, with a negative value indicating a negative interaction, a positive value indicating positive interaction, and nonsignificant difference with 0 indicating a neutral interaction.

In order to estimate the effects of drought stress, mowing disturbance, and neighbor species on each target species’ RII of tiller number (biomass), general linear model (GLM) was used with RII as dependent variables, drought stress, mowing treatment and neighbor species as fixed factors. If there were significant effects of the main factor(s) and no significant interaction between main factors, independent‐sample test or one‐way ANOVA was used to measure the effect of main factor(s) on the variables. In addition, one‐sample *t* test was applied to assess the difference between each species’ RII of tiller number (biomass) under each treatment and 0. Analysis was carried out in R (3.1.1, www.r-project.org), and the figures were created with Sigmaplot 12.5.

## 3 RESULTS

### 3.1 *S. grandis*’ RII of tiller number and biomass

**Figure 1:**
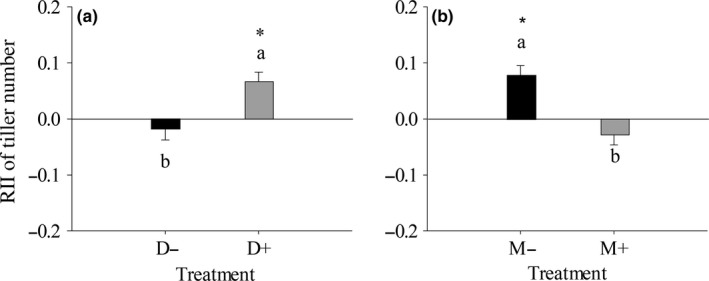
One‐way ANOVA of drought (a) and mowing (b) on *Stipa grandis*’ RII of tiller number. The different lowercase letters represent significant difference between treatments at 0.05 level. The asterisk represents significant difference between the average value of RII and 0 at 0.05 level

**Figure 2:**
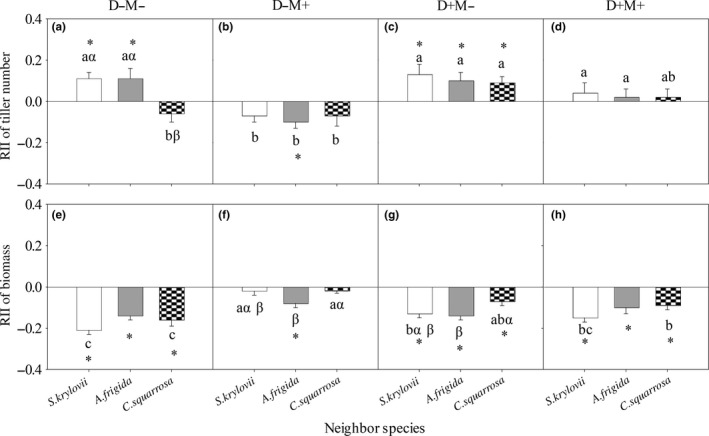
*Stipa grandis*’ RII of tiller number (a, b, c, and d) and of biomass (e, f, g, and h) with different neighbor species under each treatment. D−M−: nondrought and un‐mowing; D−M+: nondrought and mowing; D+M−: drought and un‐mowing; D+M+: drought and mowing. The same lowercase letters within the same neighbor species represent nonsignificant difference among treatments. The same Greek letters within the same treatment represent nonsignificant difference among different neighbor species. The asterisk represents significant difference between the average value of RII and 0

The overall effects of drought (D) and mowing (M) significantly (*p < *0. 05) influenced *S. grandis*’ RII of tiller number (Table 1, Figure 1). Under drought treatment (D+), the RII of tiller number was significantly (*p < *0. 05) higher than 0 and higher than that under non‐drought treatment (D−) (Figure 1a). Under un‐mowing treatment (M−), it was significantly (*p < *0. 05) higher than 0 and higher than that under mowing treatment (M+) (Figure 1b). In detail, *S. krylovii* had positive effects on *S. grandis*’ RII of tiller number under D−M− and D+M− treatments (Figure 2a, 2c); *A. frigida* had positive effects under D−M− and D+M− treatments (Figure 2a, 2c), but a negative effect under D−M+ treatment (Figure 2b); and *C. squarrosa* had a positive effect under D+M− treatment (Figure 2c).

**Table 1:** Results of general linear model for *Stipa grandis’* RII of tiller number and biomass

Source of variation	Relative interaction index (RII) of tiller number			Relative interaction index (RII) of biomass		
	MS	F	*p*	MS	F	*p*
Drought (D)	0.148	12.799	**0.001**	0.002	0.486	0.488
Mowing (M)	0.235	20.364	**<0.001**	0.088	24.877	**<0.001**
Neighbor species (N)	0.023	1.999	0.143	0.013	3.741	**0.028**
D × M	0.016	1.390	0.242	0.083	23.435	**<0.001**
D × N	0.009	0.762	0.471	0.003	0.739	0.481
M × N	0.022	1.908	0.156	0.003	0.710	0.495
D × M × N	0.018	1.550	0.219	0.014	3.971	**0.023**

The *p* values that are lower than 0.05 are in bold type.

Mowing (M), neighbor (N), D × M, and D × M × N significantly (*p < *0.05) influenced *S. grandis*’ RII of biomass (Table 1). In detail, *S. krylovii* and *C. squarrosa* had negative effects on *S. grandis*’ RII of biomass under D−M−, D+M−, and D+M+ but not D−M+ treatment; and *A. frigida* had a negative effect under each of the four treatments (Figure 2e–h).

### 3.2 *S. krylovii*'s RII of tiller number and biomass

**Figure 3:**
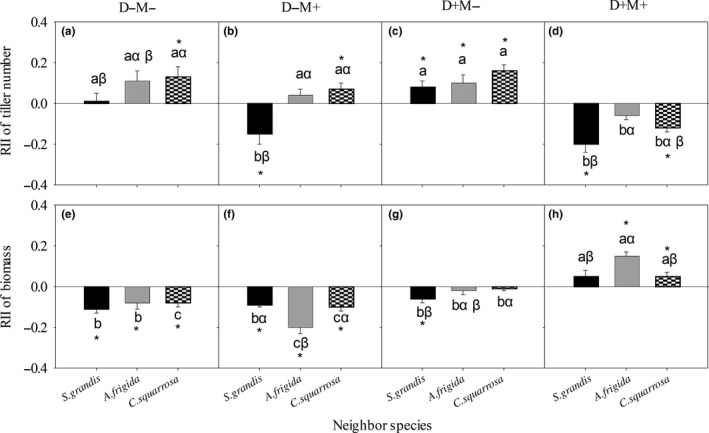
*Stipa krylovii's* RII of tiller number (a, b, c, and d) and of biomass (e, f, g, and h) with different neighbor species under each treatment. D−M−: nondrought and un‐mowing; D−M+: nondrought and mowing; D+M−: drought and un‐mowing; D+M+: drought and mowing. The same lowercase letters within the same neighbor species represent nonsignificant difference among treatments. The same Greek letters within the same treatment represent nonsignificant difference among different neighbor species. The asterisk represents significant difference between the average value of RII and 0 at 0.05 level

The overall effects of N, M, D × M, and M × N were significant (*p* < 0. 05) on *S. krylovii*'s RII of tiller number (Table 2). *S. grandis* had significantly (*p < *0. 05) negative effects on *S. krylovii*’s RII of tiller number under D−M+, D+M+ treatments, but a positive effect under D+M− treatment (Figure 3b–d); *A. frigida* had a positive effect under D+M− treatment (Figure 3c); and *C. squarrosa* had positive effects under D−M−, D−M+, and D+M− but not D+M+ treatment (Figure 3a–d).

**Table 2:** Results of general linear model for *Stipa krylovii*'s RII of tiller number and biomass

Source of variation	Relative interaction index (RII) of tiller number			Relative interaction index (RII) of biomass		
	MS	F	*p*	MS	F	*p*
Drought (D)	0.009	1.129	0.292	0.392	126.371	**<0.001**
Mowing (M)	0.523	62.658	**<0.001**	0.028	8.858	**<0.001**
Neighbor species (N)	0.117	13.955	**<0.001**	0.002	0.713	0.493
D × M	0.178	21.349	**<0.001**	0.127	40.745	**<0**.**001**
D × N	0.019	2.294	0.108	0.025	8.104	**0.001**
M × N	0.030	3.575	**0.033**	0.005	1.638	0.201
D × M × N	0.005	0.598	0.552	0.025	8.040	**0.001**

The *p* values that are lower than 0.05 are in bold type.

Mowing (M), drought (D), D × M, D × N, and D × M × N had significant (*p < *0. 05) effects on *S. krylovii*'s RII of biomass (Table 2). *S. grandis* had negative effects on *S. krylovii*'s RII of biomass under D−M−, D−M+, and D+M− but not D+M+ treatment (Figure 3); *A. frigida* had a positive effect under D+M+ treatment (Figure 3h), but negative effects under D−M− and D−M+ treatments (Figure 3e, 3f); and *C. squarrosa* had negative effects under D−M− and D−M+ treatments but a positive effect under D+M+ treatment (Figure 3e–h).

### 3.3 *A. frigida*'s RII of tiller number and biomass

**Figure 4:**
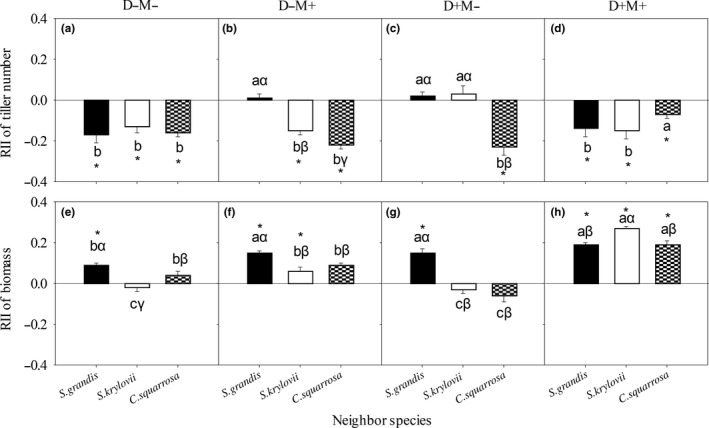
*Artemisia frigida's* RII of tiller number (a, b, c, and d) and of biomass (e, f, g, and h) with different neighbor species under each treatment. D−M−: nondrought and un‐mowing; D−M+: nondrought and mowing; D+M−: drought and un‐mowing; D+M+: drought and mowing. The same lowercase letters within the same neighbor species represent nonsignificant difference among treatments. The same Greek letters within the same treatment represent nonsignificant difference among different neighbor species. The asterisk represents significant difference between the average value of RII and 0 at 0.05 level

The overall effects of N, D × M, M × N, and D × M × N were significant (*p < *0. 05) on *A. frigida*'s RII of tiller number. *S. grandis* had negative effects on *A. frigida*'s RII of tiller number under D−M−, D+M+ treatments (Figure 4a, 4d); *S. krylovii* had negative effects under D−M−, D−M+, and D+M+ treatments (Figure 4a, 4b, 4d); and *C. squarrosa* had a negative effect under each of the four treatments (Figure 4a–d).

Mowing (M), drought (D), neighbor species (N), D × M, M × N, D × N, and D × M×N had significant (*p < *0.05) effects on *A. frigida*'s RII of biomass (Table 3). *S. grandis* had a positive effect on *A. frigida*'s RII of biomass under each of the four treatments (Figure 4e–h); *S. krylovii* had positive effects under D−M+ and D+M+ treatments (Figure 4f, 4h); and *C. squarrosa* had a positive effect under D+M+ treatment (Figure 4h).

**Table 3:** Results of general linear model for *Artemisia frigida*'s RII of tiller number and biomass

Source of variation	Relative interaction index (RII) of tiller number			Relative interaction index (RII) of biomass		
	MS	F	*p*	MS	F	*p*
Drought (D)	0.043	6.103	**0.016**	0.051	24.007	**<0.001**
Mowing (M)	0.005	0.678	0.413	0.352	167.416	**<0**.**001**
Neighbor species (N)	0.073	10.420	**<0.001**	0.057	27.162	**<0.001**
D × M	0.049	7.041	**0.010**	0.088	41.854	**<0.001**
D × N	0.007	0.986	0.378	0.020	9.674	**<0.001**
M × N	0.045	6.477	**0.003**	0.035	16.599	**<0.001**
D × M × N	0.147	21.030	**<0.001**	0.031	14.807	**<0.001**

The *p* values that are lower than 0.05 are in bold type.

### 3.4 *C. squarrosa*'s RII of tiller number and biomass

**Figure 5:**
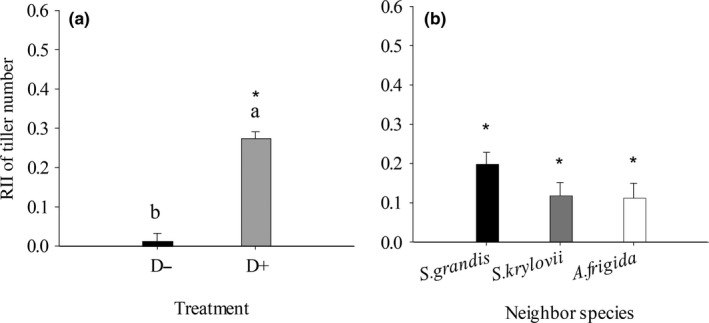
Independent‐sample test of drought (a) and one‐way ANOVA of neighbor species (b) on *Cleistogenes Squarrosa's* RII of tiller number. The different lowercase letters represent significant difference between treatments at 0.05 level. The asterisk represents significant difference between the average value of RII and 0 at 0.05 level

**Figure 6:**
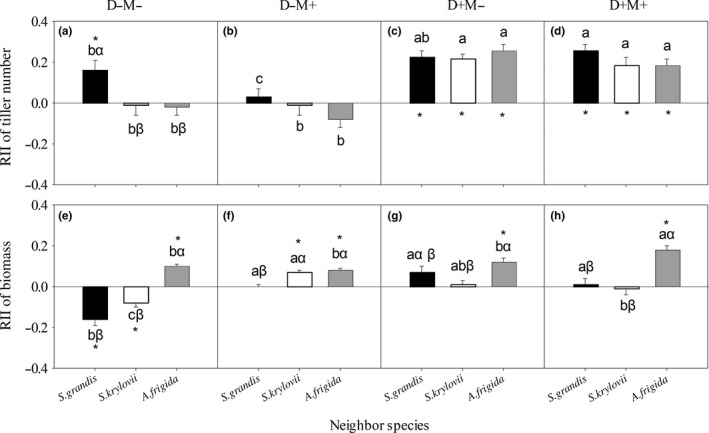
*Cleistogenes Squarrosa's* RII of tiller number (a, b, c, and d) and of biomass (e, f, g, and h) with different neighbor species under each treatment. D−M−: nondrought and un‐mowing; D−M+: nondrought and mowing; D+M−: drought and un‐mowing; D+M+: drought and mowing. The same lowercase letters within the same neighbor species represent nonsignificant difference among treatments. The same Greek letters within the same treatment represent nonsignificant difference among different neighbor species. The asterisk represents significant difference between the average value of RII and 0 at 0.05 level

The overall effects of N and D were significant (*p < *0. 05) on *C. squarrosa*'s RII of tiller number. The result of main effect analysis showed that *C. squarrosa*'s RII of tiller number under drought treatment was higher than 0 and higher than that under nondrought treatment (Figure 5a). In detail, *S. grandis* had positive effects on *C. squarrosa*'s RII of tiller number under D−M−, D+M−, and D+M+ treatments (Figure 6a–d); *S. krylovii* and *A. frigida* had positive effects under D+M− and D+M+ treatments (Figure 6c, 6d).

Mowing (M), drought (D), neighbor species (N), D × M, D × N, and D × M × N had significant (*p < *0.05) effects on *C. squarrosa*'s RII of biomass (Table 4). *S. grandis* had a negative effect on *C. squarrosa*'s RII of biomass under D−M− treatment (Figure 6e); *S. krylovii* had a negative effect under D−M− treatment, but a positive effect under D−M+ treatment (Figure 6e, 6f); and *A. frigida* had a positive effect under each of the four treatments (Figure 6e–h).

**Table 4:** Results of general linear model for *Cleistogenes squarrosa*'s RII of tiller number and biomass

Source of variation	Relative interaction index (RII) of tiller number			Relative interaction index (RII) of biomass		
	MS	*F*	*p*	MS	*F*	*p*
Drought (D)	1.454	110.644	**<0.001**	0.083	23.053	**<0.001**
Mowing (M)	0.040	3.035	0.086	0.044	12.193	**0.001**
Neighbor species (N)	0.064	4.857	**0.010**	0.153	42.539	**<0.001**
D × M	0.005	0.383	0.538	0.058	16.009	**<0**.**001**
D × N	0.024	1.795	0.174	0.022	6.151	**0.003**
M × N	0.005	0.356	0.702	0.004	1.019	0.366
D × M × N	0.027	2.019	0.140	0.044	12.116	**<0**.**001**

The *p* values that are lower than 0.05 are in bold type.

### 3.5 The comparison of the four species’ RII

**Figure 7:**
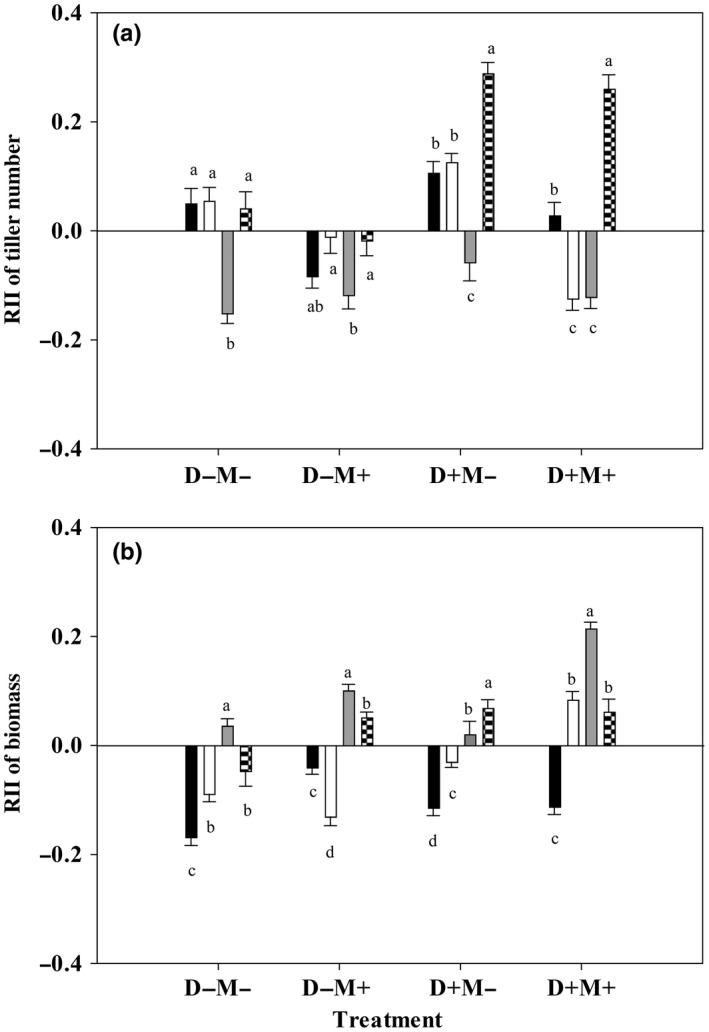
One‐way ANOVA of plant species on RII of tiller number (a) and RII of biomass (b) under the same treatment. Black bar: *Stipa grandis*; White bar: *Stipa krylovii*; Gray bar: *Artemisia frigida*; Latticed bar: *Cleistogenes squarrosa*. D−M−: nondrought and un‐mowing; D−M+: nondrought and mowing; D+M−: drought and un‐mowing; D+M+: drought and mowing. The same lowercase letters within the same treatment represent nonsignificant difference among species at 0.05 level

Under D− treatments, *A. frigida*'s RII of tiller number was significantly (*p < *0. 05) lower than any of the other three species’, and there was no significant difference among the other three species’. Under D+ treatments, *C. squarrosa'*s RII of tiller number was higher than any of the other three species’; both *S. grandis*’ and *S. krylovii*'s RIIs were higher than *A. frigida*'s under D+M− treatment, and *S. grandis*’ RII was higher than *S. krylovii*'s and *A. frigida*'s under D+M+ treatment (Figure 7a).

*A. frigida*'s RII of biomass was significantly (*p < *0. 05) higher than *S. grandis*’ and *S. krylovii*'s under each of the four treatments, and *C. squarrosa'*s RII was significantly (*p < *0. 05) higher than *S. grandis*’ under each of the four treatments and higher than *S. krylovii*'s under D−M+ and D+M− treatments (Figure 7b).

## 4 DISCUSSION

Our study revealed the reverse trends between the RIIs of tiller number and the RIIs of biomass for the same species under the same condition. That is to say, under the same condition, if target species’ RII of tiller number was positive, its RII of biomass usually was negative (Figures 1 and 5). Therefore, in the present study, we used RII of tiller number to show the competitive capacity of the three Gramineae species, and RII of biomass to show the competitive capacity of the small subshrub *A. frigida* because *A. frigida* expands by generating adventitious roots from its tillers.

Our study showed that the mowing disturbance but not drought stress was the main driver of reducing competitive capacity of *S. grandis*, which would cause the retrogressive succession of *S. grandis* communities. First, disturbance of mowing played more important role in affecting *S. grandis*’ RII of tiller number than drought stress (Figure 1). As the dominant species in the typical steppe of Inner Mongolia Steppe, *S. grandis* exhibits superior growth relative to the other species in the community, but disturbance by mowing would decrease its competitive advantage. High competitive ability and low tolerance to disturbance enable a species to minimize the costs of negative interactions and to maximize the benefits of habitat amelioration provided by neighbors (Liancourt, Callaway, & Michalet, 2005). For example, all the neighbor species showed significantly positive effects on *S. grandis*’ RII of tiller number under un‐mowing treatment, but no significant effect was found under mowing treatment (Tables 1 and 2; Figures 1 and 2). While, comparing with the RIIs under nondrought treatment, the RIIs of tiller number were higher under drought treatment. Mowed target species may suffer more under drought condition than under nondrought condition and thus may be more demanding of stress mitigation by neighbors, as shown in physically disturbed communities (Maalouf, Bagousse‐Pinguet, Marchand, Touzard, & Michalet, 2012). Second, drought increased *S. grandis*'s RII of tiller number but did not affect the counterpart of *S. krylovii* (Figures 2 and 3), indicating that drought stress could not be the main driver for the replacement of *S. grandis* by *S. krylovii*.

As the dominant species in early degradation stage of *S. grandis* communities, *S. krylovii*'s RII of tiller number was affected by mowing, neighbor species, interaction of drought and mowing. Especially, the mowing was the first and foremost factor (the MS values in Table 2) in affecting it, which could cause the replacement of *S. krylovii* by other species, such as *A. frigida* (Zhang, Li, Jiang, Lin, & Du, 2005). Furthermore, the researches about changes in competition hierarchies caused by physical environment or/and neighbor specie are one of the most attractive issues in modern ecology (Jiménez & Rossi, 2012). A few studies have manifested that neighbor species could change species competition hierarchies (Chen et al., 2013; Michalet et al., 2014). Under D−M+ treatment, *S. grandis*' RII of tiller number was negative when *S. grandis* was grown with *A. frigida* and neutral when *S. grandis* was grown with the other two species (Figure 2b), while *S. krylovii*'s RII of tiller number was negative when *S. krylovii* was grown with *S. grandis,* neutral when *S. krylovii* was grown with *A. frigida*, and positive when *S. krylovii* was grown with *C. squarrosa* (Figure 3b). These results supported the viewpoint that neighbor species could significantly affect the competitive hierarchy of the target species (Ulrich et al., 2016). Under D−M+ condition, *C. squarrosa* enhancing *S. krylovii*'s competitive capacity could provide *S. krylovii* a chance to increase its abundance, however, *A. fridida* decreasing *S. grandis*’ competitive capacity would shrink *S. grandis*’ population. Therefore, the different effects of neighbor species on *S. grandis* and *S. krylovii* may be one of the reasons for the replacement of *S. grandis* by *S. krylovii* under grazing conditions in the natural communities.

The present results indicated that mowing could increase *A. frigida*'s RII of biomass and be beneficial for *A. frigida*'s population expansion. The spreading type subshrub *A. frigida* can develop adventitious roots when its tillers touch the ground, which helps its population expansion (Yang, 2001). Therefore, *A. frigida*'s RII of biomass has more practical significance than RII of tiller number. Mowing disturbance had a positive effect on *A. frigida*'s RII of biomass (Figure 4). Combining the result that mowing disturbance inhibited *S. grandis*’ and *S. krylovii*'s RIIs, we could predict that *A. frigida* gained more benefit than *S. grandis* and *S. krylovii* from mowing disturbance. That is to say, mowing disturbance played a determination role in the dominant replacement of *S. grandis* and *S. krylovii* by *A. frigida* during the retrogressive succession. In addition, the neighbor species restricted tiller of subshrub *A. frigida* but facilitated its biomass (Figure 4). These results indicated that gap community would be beneficial to *A. frigida*'s expanding by generating plentiful adventitious root and *A. frigida* could become the dominant species in the medium–heavy‐degraded communities. And, they also suggested that *A. frigida* cannot become the dominant species in the communities with few gaps, such as the stable *S. grandis* communities or the early stage of *S. grandis* communities’ retrogressive succession (Yang, Bai, Li, & Han, 2009).

Comparing with the other three species, the stronger tolerance to drought played an important role in helping *C. squarrosa* become the dominant specie in the heavy‐degraded communities. *C. squarrosa*'s RII of tiller number was significantly affected by drought, mowing, and neighbor species, especially drought (Figure 5a, Table 4). Drought significantly increased *C. squarrosa*'s RII of tiller number, especially under drought and mowing (D+M+) treatment (Figure 6c, 6d), and *C. squarrosa*'s RIIs of tiller number were positive under drought treatment and were not influenced by neighbor species (Figure 5b). As a C_4_ plant, *C. squarrosa* has a higher photosynthetic capacity and water‐use efficiency than the C_3_ plants and a relatively higher adaptive capacity to drought (Wang, 2004; Wang, Wang, & Chen, 2003). In heavy‐degraded steppe, similar results that *C. squarrosa* could take advantage of increasing tiller and become dominant species have been reported by researchers (Qin et al., 2017; You & Yang, 2016). These results supported that the changes in growing abilities and drought‐induced habitat amelioration co‐determine the dominance of the species in the community (Hacker & Gaines, 1997).

## 5 CONCLUSIONS

The present research analyzed how the four dominant species in the retrogressive succession of the typical steppe of Inner Mongolia responded to drought, mowing, and neighbor species under different treatments in a controlled experiment. Mowing disturbance could decrease the competitive ability of *S. grandis* and *S. krylovii* but increase competitive ability of *A. frigida*, and may be the driving force in the light to medium stage of retrogressive succession. The higher drought tolerance but lower competitive ability may be the reason that *C. squarrosa* could become the dominant species just in the heavy‐degraded regions of typical steppe but not in the stable or light‐degraded regions. These results could help us to explain the retrogressive succession and to predict the community dynamics facing environmental changes in the semi‐arid steppe of China.

## CONFLICT OF INTEREST

None declared.

## AUTHORS CONTRIBUTION

Shaobo Gao wrote the manuscript and accomplished the experiment, Zhirong Zheng analyzed the data, Yukun Wang and Lei Liu took part in the experiment and collected data, Nianxi Zhao designed the experiment and revised the manuscript, and Yubao Gao revised the manuscript.

## DATA ACCESSIBILITY

Data underlying this article can be accessed on Dryad. We agree to deposit our data to a public repository.
